# Recent Advances in Cardiac Resynchronization Therapy: Current Treatment and Future Direction

**DOI:** 10.3390/jcm14030889

**Published:** 2025-01-29

**Authors:** Arsalan Siddiqui, Vasiliki Tasouli-Drakou, Marc Ringor, Michael V. DiCaro, Brianna Yee, KaChon Lei, Tahir Tak

**Affiliations:** Department of Medicine, Kirk Kerkorian School of Medicine at UNLV, Las Vegas, NV 89102, USA; vasiliki.tasoulidrakou@unlv.edu (V.T.-D.); marc.ringor@unlv.edu (M.R.); michael.dicaro@unlv.edu (M.V.D.); brianna.yee@unlv.edu (B.Y.); kachon.lei@unlv.edu (K.L.); tahir.tak@va.gov (T.T.)

**Keywords:** cardiac resynchronization therapy, cardiac physiologic pacing, conduction system pacing, heart failure, His-bundle pacing, Left bundle branch area pacing, cardiac contractility modulation

## Abstract

Cardiac Resynchronization Therapy (CRT) has been established as a major component of heart failure management, resulting in a significant reduction in patient morbidity and death for patients with increased QRS duration, low left ventricular ejection fraction (LVEF), and high risk of arrhythmias. The ability to synchronize both ventricles, lower heart failure hospitalizations, and optimize clinical outcomes are some of the attractive characteristics of biventricular pacing, or CRT. However, the high rate of CRT non-responders has led to the development of new modalities including leadless CRT pacemakers (CRT-P) and devices focused on conduction system pacing (CSP). This comprehensive review aims to present recent findings from CRT clinical trials and systematic reviews that have been published that will likely guide future directions in patient care.

## 1. Introduction

Heart failure (HF) affects more than 64 million people worldwide, imposing a tremendous burden on society, both socially and economically [1]. A leading cause of mortality in HF patients is sudden cardiac death (SCD), often attributed to the development of electrical abnormalities [2]. Due to structural and functional changes occurring in HF patients over time, these patients are predisposed to developing significant conduction disease, including atrioventricular (AV) dissociation, premature ventricular contractions (PVCs), atrial fibrillation (AF), ventricular dyssynchrony, and loss of the atrial kick [3,4,5]. These pathologic changes, if left untreated, contribute to contractile dysfunction, reduced cardiac output, and malignant arrhythmias, which can be fatal [6]. As such, evidence-based treatment and prevention strategies for HF patients are crucial. To mitigate the risk of SCD, avoid cardiac remodeling, and improve ventricular function, innovative treatments directly targeting the conduction system, such as Cardiac Resynchronization Therapy (CRT) have been developed.

CRT can utilize either a pacemaker (CRT-P) or a pacemaker-defibrillator (CRT-D), with electrical lead placement through the coronary sinus (CS) to the conduction centers. While both devices deliver impulses to synchronize atrial and ventricular contraction, CRT-D is more commonly implanted in heart failure (HF) patients with an EF < 35%, as it provides the added benefit of defibrillation for arrhythmia prevention [7]. Since its introduction, many studies have been conducted to determine the efficacy of CRT on patients with HF, with many of these demonstrating short and long-term benefits on morbidity, mortality, and quality of life [8]. Moreover, patients undergoing CRT have demonstrated reductions in HF symptoms and hospitalizations, with some also showing evidence of reverse remodeling in the left ventricle [9]. Nonetheless, a great percentage of patients (20–40%) fail to respond to CRT. Subsequent studies have sought to enhance the effectiveness of treatment by improving rate control, synchronicity, and cardiac performance, leading to new innovations in application and technology, such as echocardiogram-assisted coordination in ventricular synchronization and leadless pacemakers.

Given the evolving landscape of CRT, this review aims to provide a comprehensive analysis of its current applications, advancements, and limitations. This review will delve into the mechanisms underpinning CRT, its clinical efficacy, emerging innovations such as leadless systems and conduction system pacing, and challenges associated with patient selection and non-responder management. By synthesizing the best available evidence, this paper seeks to outline the future directions of CRT and its potential to improve HF management.

## 2. Methods

A literature search was conducted across multiple bibliographic databases, including PubMed, MEDLINE, and Google Scholar. The primary questions to answer for this comprehensive review were “What is the current best available evidence for the clinical application of cardiac resynchronization therapy” and “What are some novel recent advances in cardiac resynchronization therapy”. Given that the most recent 2022 HF guidelines published in conjunction by the American Heart Association (AHA), American College of Cardiology (ACC), and Heart Failure Society of America (HFSA) incorporated reviews until December 2020 and clinical trials through September 2021, the date range for publications was adjusted with this in mind to include relevant literature published thereafter [10]. The phrase encompassing the keywords “cardiac resynchronization therapy”, “heart failure”, and “cardiac physiologic pacing” was used in this search. Article types included clinical trials, randomized controlled trials, literature reviews, systematic reviews, and meta-analyses. For systematic reviews and meta-analyses, the publication date range was set from 1 January 2021 to 6 November 2024. The clinical and randomized control trials date range was set from 1 October 2021 to 6 November 2024. Several articles were obtained from previously referenced publications, and the most recent and relevant articles were assessed.

## 3. Current Guidelines and Recommendations

The New York Heart Association (NYHA) functional classification subdivides HF patients into four classes: Class I denotes patients with cardiac disease but without resulting limitation of physical activity, classes II and III describe patients with cardiac disease resulting in slight and marked limitation of physical activity, respectively, and class IV encompasses patients with HF resulting in inability to engage in any physical activity without discomfort [11].

In patients with HF, CRT eligibility is determined largely in conjunction with the NYHA classification mentioned above, in addition to considering if patients have undergone maximally tolerated guideline-directed medical therapy (GDMT). Key medication classes of GDMT include Mineralocorticoid Receptor Antagonists, Sodium–Glucose Cotransporter-2 Inhibitors, Beta blockers, and Angiotensin receptor blockers/Angiotensin Converting Enzyme Inhibitors/Angiotensin receptor/Neprilysin Inhibitors. Additionally, hydralazine-nitrates are also considered GDMT in specific populations. These medications target the adverse neurohormonal activation pathways due to HF and have demonstrated significant benefits in reducing left ventricular remodeling, morbidity, and hospitalizations [10]. If, after a trial of GDMT does not lead to interval improvement in terms of functional status or EF, then CRT is considered. The AHA and ESC recommend a duration of 3 months of GDMT prior to consideration of CRT implantation [10,12].

**Figure 1:**
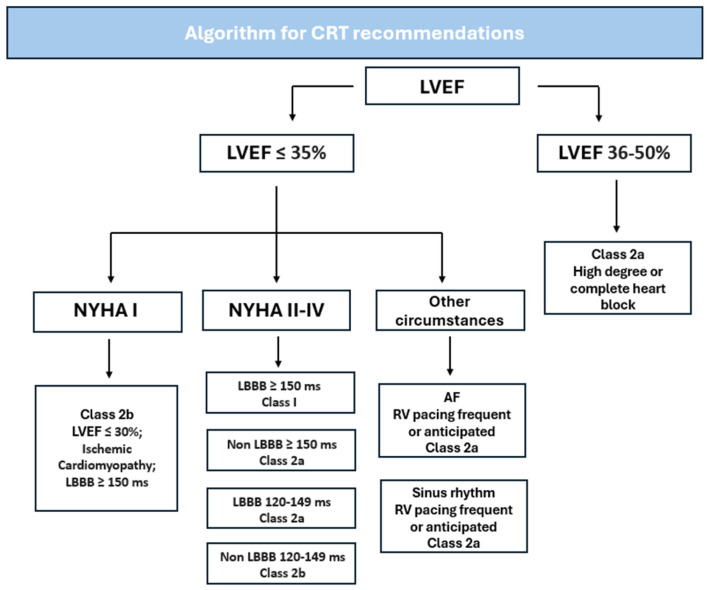
Algorithm for CRT Indications Proposed by the 2022 AHA/ACC/HFSA Guidelines For the Management of Heart Failure. LVEF: Left Ventricular Ejection Fraction; NYHA: New York Heart Association; LBBB: Left Bundle Branch Block; AF: Atrial Fibrillation; RV: Right ventricle.

Per the most recent 2022 guidelines, jointly published in conjunction with the AHA, ACC, and HFSA, CRT is indicated for patients with an LVEF equal or less than 35%, sinus rhythm, left bundle branch block (LBBB) with a QRS duration (QRSd) equal or greater than 150 ms, and NYHA Class II, III or ambulatory class IV symptoms already on GDMT [10]. CRT may also be useful in patients with concomitant AF and LVEF equal to or less than 35%, patients with a high-degree heart block and LVEF of 36% to 50%, patients with genetic arrhythmogenic cardiomyopathy and LVEF equal to or less than 45%, patients with an LVEF equal to or less than 35%, sinus rhythm, LBBB or non-LBBB pattern with a QRSd between 120 and 149 ms, and NYHA Class III or ambulatory class IV on GDMT. At this time, the 2022 guidelines do not recommend CRT in patients with QRS less than 120 ms [10]. In contrast, patients whose QRSd is not increased to suggest ventricular dyssynchrony and who may still present with an LVEF <35% and NYHA class II or III, or with possible documented ventricular fibrillation or hemodynamically unstable sustained ventricular tachycardia, may be recommended to receive an implantable cardioverter-defibrillator (ICD) [13]. These guidelines are summarized in Table 1 and Figure 1 below.

Similarly, the 2021 European Society of Cardiology also provides recommendations for the use of CRT in HF management; however, they have a slight variance compared to the AHA guidelines13. They provide class 1 recommendations in HF patients with LVEF equal or less than 35%, sinus rhythm, LBBB with a QRSd equal or greater than 150 ms as well as in HF patients with reduced EF (HFrEF) who have indications for ventricular pacing for high-degree AV block regardless of QRSd or NYHA classification. Class 2a recommendations include consideration of CRT in patients with LVEF less than 35%, sinus rhythm, non-LBBB pattern with QRSd equal or greater than 150 ms, in patients with LVEF less than 35%, sinus rhythm, and LBBB pattern with QRS 130 to 149 ms in duration, as well as in those with LVEF equal or less than 35%, previously implanted ICD, and subsequently worsening HF symptoms. Class 2b recommendation includes consideration of CRT for symptomatic patients with LVEF equal or less than 35%, sinus rhythm, non-LBBB with a QRSd between 130 and 149 ms.

### 3.1. Goals for CRT

The close link between electrical excitation and mechanical contraction/relaxation has been well established, both in patients with healthy cardiac function as well as those with HF [14,15,16,17]. Due to HF-related pathologic electromechanical changes, this can result in ventricular dyssynchrony or uncoordinated contraction of the left and right ventricle [18,19]. This insufficiency, in turn, can result in reduced left ventricular contractility, increased mitral regurgitation, negative septal wall motion, and poor clinical outcomes, including increased risk of morbidity and mortality [18,20,21,22]. Therefore, the purpose of CRT is to help restore coordinated electrical activity and improve ventricular synchrony. If this is achieved, it has been shown to improve cardiac output, reduce mitral regurgitation, reverse HF-related cardiac remodeling, and improve clinical status [23].

### 3.2. Measured Outcomes for CRT

Measured outcomes used to evaluate CRT effectiveness vary amongst studies, with no definitive consensus on what constitutes adequate response. The 2023 HRS/APHRS/LAHRS guidelines on cardiac physiologic pacing for the avoidance and mitigation of HF suggest both clinical and echocardiographic criteria to determine the adequacy of response [24]. Clinical criteria include a measured reduction in mortality, HF hospitalization, NYHA class improvement, improvement in 6 min walk test, reduction in HF medications, and increase in peak VO2 [24]. Echocardiographic markers include improvement or stability in LVEF, reduction in left ventricular size, increased left ventricular stroke volume, and/or reduction in mitral regurgitation [24]. While not mentioned in the aforementioned guidelines, an important electrocardiographic metric to measure the success of CRT includes QRSd and morphology. Due to the mechanical and electrical dyssynchrony as a result of cardiac remodeling in HF, the QRSd provides an objective parameter for intraventricular delay. Studies have previously shown that a greater decrease in QRSd is associated with improved left ventricular reverse remodeling, HF hospitalizations, and death [25,26]. Given this, the AHA denotes QRSd as part of the indication criteria for CRT [10].

## 4. Types of CRT

**Figure 2:**
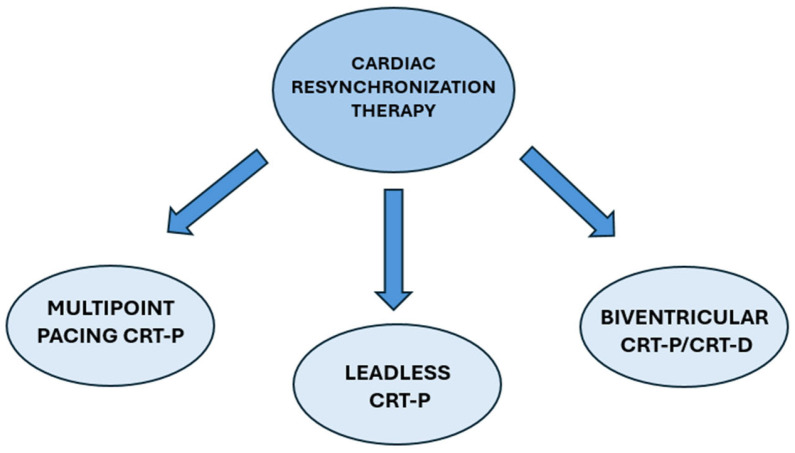
Summary of the different types of CRT devices. CRT: Cardiac Resynchronization Therapy; CRT-P: Cardiac Resynchronization Therapy with pacing activity; CRT-D: Cardiac Resynchronization Therapy with Defibrillator activity.

CRT-P consists of a pacemaker which typically has two components: a pulse generator, which produces the electrical activity that will be transmitted to the myocardium, and electrodes (colloquially known as leads) which are responsible for transmitting the electrical activity [27]. CRT can be further classified based on electrode type/placement, including biventricular, leadless, and multipoint pacing. Certain CRT devices can also offer defibrillation capabilities (known as CRT-D), with questionable superiority over conventional CRT pacemakers [28]. A summary of CRT device types is shown in Figure 2.

### 4.1. Biventricular CRT

**Figure 3:**
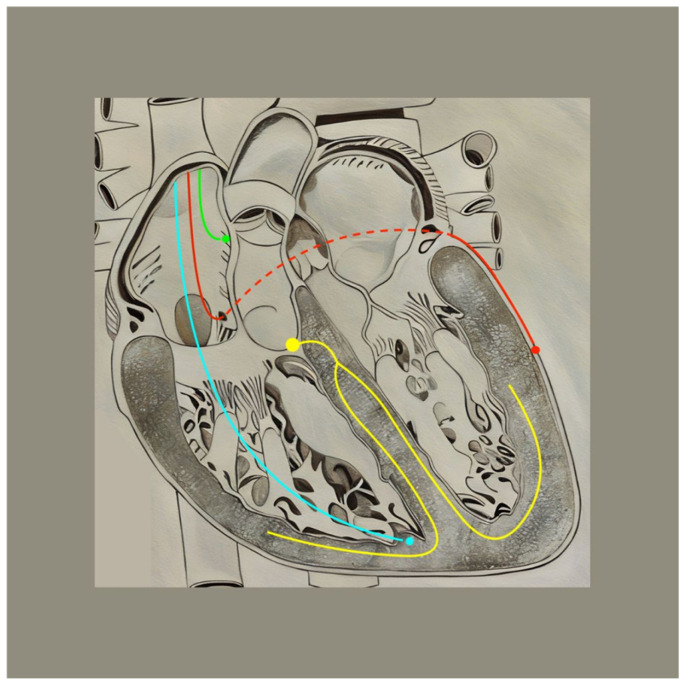
Lead positioning in BiV-CRT. Blue = Right Ventricular Lead; Red = Left ventricular lead; Green = Right atrial lead; Yellow = Intrinsic His–Purkinje system.

Biventricular CRT (BiV-CRT) is the most studied and well-established CRT modality in the management of HF after results from several landmark trials such COMPANION, MIRACLE, REVERSE, MADIT-CRT, CARE-HF, and RAFT, as referenced in the 2022 AHA guidelines on HF [10,29,30,31,32,33,34]. These trials are summarized in Table 2 below. Typically, BiV-CRT involves the placement of three leads: one in the right atrium (RA) which senses and/or paces atrial activity, and one lead each in the LV and right ventricle (RV) for pacing (Figure 3) [35]. This device causes simultaneous pacing of both ventricles, leading to improved ventricular synchrony. Implantation of BiV-CRT requires transvenous pacing of the LV via the CS, with the cephalic vein most commonly used as the point of device entry. Although the LV lead can be placed either posterolateral or anteriorly, more recent studies have shown that placement of the LV lead in posterolateral non-apical regions is associated with better CRT responses due to pacing of the latest activated sites of the LV [36]. Similarly, the RA lead is typically placed in the mid-to-upper RA for optimal signal detection and stability [37]. Furthermore, the RV septal wall (divided into three sections: apex, mid septum, and upper septum) is typically used for the placement of the RV lead, with the apex being used most frequently. However, Carpio et al. have stipulated that RV lead placement in the upper septum near the outflow tract and LV placement in the mid posterior wall result in a shorter QRSd, a favorable prognostic marker in HF patients [38].

### 4.2. Multipoint Pacing CRT

Multipoint pacing CRT (MPP-CRT) involves the placement of three leads similar to conventional BiV-CRT devices; however, the left ventricular lead is quadripolar and can pace two or more sites on the left ventricle [39]. The innovation of MPP-CRT was based on the hopes of improved contractility and synchrony when comparing it to conventional BiV-CRT. This idea was supported by a 2022 randomized clinical trial of 142 subjects that showed that at the 6-month follow-up, MPP-CRT patients had a statistically significant improvement of EF, end-systolic volume (ESV) response rate and NYHA class, as well as greater ESV reduction when compared to BiV-CRT recipients [40]. In contrast, a 2021 systematic study by Mehta et al. found that MPP-CRT, when compared to BiV-CRT, was not effective in randomized studies, but was efficacious in non-randomized studies, suggesting that the positive results could be due to the considerable heterogeneity in study designs [41]. A 2023 clinical trial by Leclercq et al. of 644 BiV-CRT patients deemed as non-responders after 6 months of original device therapy and randomly assigned to receive either MPP-CRT or continue with BiV-CRT, also found that MPP-CRT did not improve CRT response in non-responders when compared to BiV-CRT after an additional 6 months of therapy [42]. Hence, larger randomized trials comparing MPP-CRT with BiV-CRT are needed.

### 4.3. CRT-Defibrillators

CRT devices with defibrillation capabilities offer additional protection against SCD, particularly in high-risk patients [43]. Factors that are associated with SCD include an early repolarization pattern (ERP) on ECG, type 2 diabetes mellitus, AF, hypertension, cigarette smoking, and to a lesser extent left ventricular hypertrophy, nonsustained ventricular tachycardia and reduced LVEF [44].

A systematic review of seven observational studies and one RCT comparing CRT-Ps with CRT-Ds in individuals with nonischemic cardiomyopathy (NICM) found that the addition of defibrillator therapy was not significantly associated with a reduction in all-cause mortality in CRT-eligible patients [45]. This finding was corroborated by a meta-analysis conducted by Long et al., which evaluated 21 studies and 69,919 patients and found no significant difference in all-cause mortality among patients with nonischemic cardiomyopathy (NICM) or those aged over 75 years [46]. However, their meta-analysis indicated that there was a significant reduction in all-cause mortality in individuals with ischemic cardiomyopathy (ICM). These discrepancies may be explained by the fact that ICM patients have a higher risk of death from fatal arrhythmias compared to NICM individuals, as NICM patients exhibit a greater LV systolic function and LV reverse remodeling.

Outcome improvement with CRT-D is found particularly in comparison to ICDs. Recent studies trend toward a reduction in mortality in patients with CRT-D devices compared to ICDs. A meta-analysis by Liu et al. that evaluated individuals with chronic kidney disease (CKD) and renal insufficiency found that the risk of all-cause mortality was lower in CKD patients with a CRT-D than those with an ICD, while taking into consideration that CKD class 4 and 5 individuals had a higher risk of all-cause mortality compared to patients with CKD class 3 [47]. Moreover, an RCT by Sapp et al. examined the long-term survival of 1050 patients who were either assigned to receive an ICD or a CRT-D and found that time until death appeared to be longer in the CRT-D group when looking at death from any cause [48]. Hence, the increasing evidence supporting long-term survival benefits of CRT-Ds by meta-analyses and RCTs may highlight the possible future shift towards the implementation of CRT-Ds over conventional ICDs. Nonetheless, more studies will be needed to evaluate additional secondary outcomes, such as improvement in NYHA classes and LVEF with CRT-D devices.

### 4.4. Image Guided CRT Placement

Given that the rate of non-responses has been associated with malpositioned lead placement, advancements in CRT have been focused on image guidance for CRT placement. In recent years, various imaging modalities such as echocardiography, cardiac magnetic resonance imaging (CMR), single-photon emission computed tomography (SPECT), and speckle tracking ECHO (STE) are being studied to optimize left ventricular-lead placement. A meta-analysis of four RCTs with an average follow-up of 2 years found that image-guided left ventricular-lead placement (mostly either in the mid-left ventricle or the anterolateral segment) was associated with significant improvements in NYHA classification by one class or greater, as well as improvement in mortality and HF hospitalization outcomes, but without an improvement in LVEF [49]. However, a larger meta-analysis of eight randomized trials and prospective studies (1075 patients) showed a statistically significant improvement in LVEF in the image-guided group [50]. Of the eight studies included, multiple imaging modalities were used, including STE, intracardiac echocardiogram coupled with vector velocity imaging, CMR, SPECT, and fusion of fluoroscopy images with CMR.

A systematic review examining the role of CMR in identifying appropriate candidates for CRT found that CMR-guided lead placement was associated with improved clinical outcomes and response to CRT due to lead placement away from areas of transmural scar [51]. On the contrary, a prospective RCT of 172 patients comparing conventional implantation with speckle tracking radial strain imaging (STRSI)-guided left ventricular lead placement supported the finding that the latter did not improve clinical or echocardiographic responses [52]. Moreover, outcomes from an RCT comparing multimodality imaging guidance to routine fluoroscopy-guided CRT implantation found that multimodality imaging guidance did not reduce HF hospitalization or all-cause death [53]. Thus, future research should identify which imaging modality improves clinical outcomes when compared to others, but also compare its outcomes with those identified from the use of multimodality imaging guidance.

In recent years, an area of growing interest has been cardiac computed tomography (CT) in guiding left ventricular-lead placement. In a study by Behar et al., 18 patients underwent CRT upgrades using a novel algorithm with cardiac CT-guided findings to identify the optimal left ventricular-lead placement [54]. The study reported improved hemodynamic and composite clinical response rates compared to their prior status [54]. Similarly, Gould et al. found that intraprocedural integration of cardiac CT is achievable in the placement of left ventricular leads away from identified scarred areas leading to improved volumetric response rates, especially in patients with prior ICM [55].

### 4.5. Remote Monitoring in CRT

Remote monitoring transmits CRT device data to healthcare providers, enabling them to assess a patient’s clinical status without direct evaluation or communication. In a cohort study involving 900 patients, Boehmer et al. demonstrated that data detected from CRT-D devices including heart sounds, respirations, and thoracic impedance could be incorporated into a validated alert algorithm that was able to determine HF exacerbations with 70% sensitivity [56]. Similarly, the SELENE-HF study employed an algorithm that integrated temporal trends including heart rate variability, physical activity, thoracic impedance, and arrhythmia burden to predict two-thirds of post-implant HF hospitalizations [57]. Further studies utilizing remote monitoring of devices have demonstrated its potential to reduce emergency hospital visits, accelerate decision-making, shorten hospitalization days, and decrease healthcare utilization costs [58,59,60,61]. Over the past 5 years, additional studies have substantiated its utility. For example, a randomized pragmatic trial in Portugal demonstrated that remote monitoring reduced the burden of in-office visits while achieving superior satisfaction among patients and providers [62].

Recently, studies integrating machine learning algorithms with remote monitoring data further suggest the significant prognostic value of remote monitoring. A post hoc analysis of the IMPACT trial by Ginder et al. found that remote monitoring data may detect malignant ventricular arrhythmias 30 days prior to device implantation and can guide appropriate device therapy [63]. The authors utilized neural networking models in their study, highlighting the potential of machine learning to enhance the prediction and management of such arrhythmias. Thus, remote monitoring may help guide the management of patients to improve outcomes, reduce in-office visit burden, and effectively avoid adverse outcomes and hospitalizations. These studies suggest that remote monitoring will confer significant potential to enhance CRT patient care in the coming years.

### 4.6. Drawbacks of CRT—Non-Response and Other Complications

While CRT has been integral in management of HF patients, there are still significant drawbacks with its usage. Most notably is ‘non-response’, which is reportedly to occur in approximately a third of CRT patients [64]. Paralleling the lack of concise definition to what is termed as CRT ‘response’, non-response usually refers to a lack of improvement (or worsening) in outcomes typically measured in CRT patients, either via clinical and/or imaging parameters [65,66]. Factors associated with suboptimal CRT response include suboptimal left ventricular lead position, arrhythmias, suboptimal medical therapy, underlying narrow QRS, persistent mechanical dyssynchrony, and primary right ventricular dysfunction [67]. Indeed, non-response to BiV-CRT remains a significant challenge and may result from direct myocardial stimulation bypassing the His–Purkinje system, variability in myocardial characteristics, or coronary sinus anatomical constraints [68]. Moreover, Varma et al. hospitalization and mortality rates were significantly higher in non-response patients compared to CRT responders [69].

In addition to non-response, CRT use may be associated with procedural complications, including lead malfunction, device infection, hematoma, and device malfunction (Table 3 below) [70,71]. These complications may occur at increased rates with CRT-D devices, which are typically larger, have less battery longevity, are more prone to erosions, and are associated with more frequent clinic visits and hospitalizations compared to CRT-P devices [72,73]. In particular, device-related infection (DRI) confers a significant burden and is associated with higher rates of hospitalizations and financial costs [74,75]. While the true incidence rate is uncertain, a 16-year trend of the Nationwide Inpatient Sample found a 1.61% incidence rate of DRI between 1993 and 2008 [76]. Similarly, between 1982 and 2018, the lifetime risk of DRIs was 2.18% for CRT-P and 3.35% for CRT-D devices in the Danish Pacemaker and ICD Registry [77]. Management of DRIs is associated with worsened outcomes. In a long-term outcomes retrospective study by Arabia et al., patients who had device removal due to infection had a mortality rate of 30% compared to 9.5% in non-infected patients [78]. Therefore, patients presenting with signs/symptoms of infection, endocarditis, or bacteremia in the setting of an implanted CRT device should be promptly evaluated for such an infection, and clinicians should remain vigilant for such a complication in this population.

## 5. Alternatives to CRT: Conduction System Pacing

**Figure 4:**
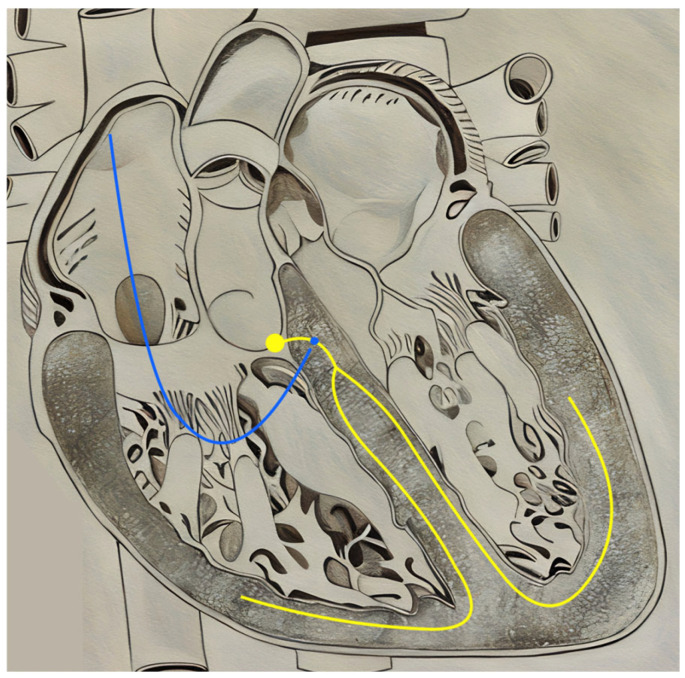
Lead positioning in His-Bundle Pacing. Blue = His-bundle Lead; Yellow = Intrinsic His–Purkinje system.

Despite the prominence of CRT as a mainstay of cardiac pacing, the rates of non-response and limitations in stimulating diseased and/or scarred myocardium have prompted interest in conduction system pacing (CSP) [71,79]. CSP involves the direct pacing of the intrinsic cardiac conduction system, specifically at the level of the His bundle and left bundle branch area (Figure 4). Proponents of these methods postulate that the intrinsic, endocardial delivery system that CSP provides may circumvent the obstacles faced by the extrinsic excitation offered by BVP-CRT. Over the past 10 years, many observational studies have found CSP to result in highly sufficient resynchronization and a feasible alternative to BiV-CRT [80,81,82,83,84,85]. The two main classifications of CSP, His-bundle pacing (HBP), and left bundle branch area pacing (LBBAP), are discussed below.

### 5.1. His-Bundle Pacing

Recent clinical trials and systematic reviews have compared the efficacy of HBP to BiV-CRT, examining clinical and imaging outcomes. A meta-analysis by Junior et al. involving 774 patients demonstrated QRSd shortening, improved LVEF, and lower NYHA classification at follow-up in patients treated with HBP compared to BVP-CRT [86].

Another randomized control trial, the HOT-CRT trial, compared BiV-CRT and HBP among patients with an LVEF < 50%, to explore which modality led to a greater change in LVEF [87]. Results showed a significantly higher number of patients experienced improved LVEF among those receiving HBP when compared to those who received BiV-CRT (80% vs. 61%, respectively) [87]. The ALTERNATIVE-AF trial compared physiologic and clinical outcomes of HF patients who received either BiV-CRT or HBP after treatment of AF post-AV nodal ablation [88]. Although many of the results, such as NYHA functional re-classification and B-type natriuretic peptide (BNP) levels, were similar between both therapies, HBP displayed slightly more improvement in LVEF, ventricular functioning, and QRSd. These studies highlight the growing interest in CSP as a potentially viable alternative to BiV-CRT.

Another potential area of HBP intervention includes patients with QRSd ≤ 140 ms. The HOPE-HF trial was a double-blind, cross-over trial including patients with HF, LVEF ≤ 40%, PR interval ≥ 200 ms, and either QRSd ≤ 140 ms or right bundle branch block [89]. Patients either received pacing with HBP or no pacing, and the study revealed a significant improvement in quality of life based on the Minnesota Living With Heart Failure Questionnaire. It also found that a majority of patients preferred pacing compared to non-pacing. A comparison of BiV-CRT and HBP can be seen in Table 4.

### 5.2. Left Bundle Branch Area Pacing

**Figure 5:**
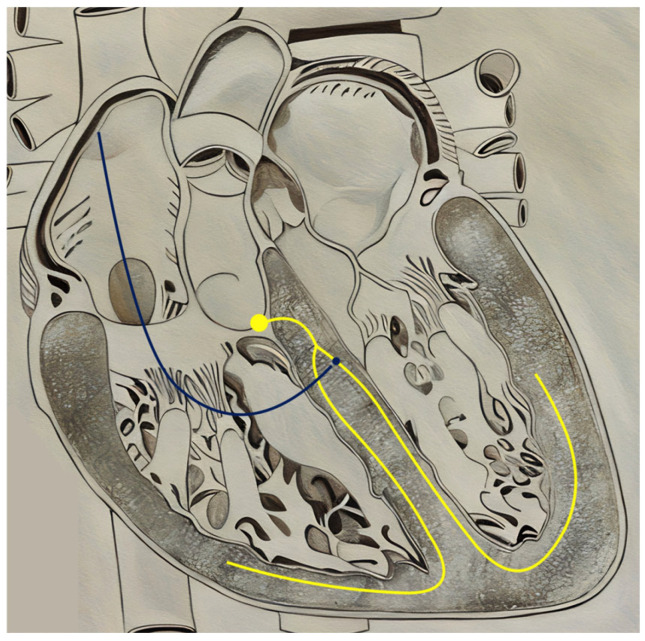
Lead positioning in Left Bundle Branch Area Pacing. Blue = Left bundle branch area lead; Yellow = Intrinsic His–Purkinje system.

Recent systematic reviews have highlighted the therapeutic potential of LBBAP (Figure 5). A review involving 772 patients from 15 observational studies found that LBBAP was associated with improved cardiac function and ventricular synchrony [90]. This improvement in synchronization theoretically improves patient outcomes, as shown by a meta-analysis involving 1063 patients that demonstrated a subclassification of LBBAP called, left bundle branch pacing (LBBP), resulting in less HF hospitalizations compared to BiV-CRT [91]. A pivotal clinical trial by Chen et al., which included 145 CRT non-responders despite optimal medical therapy and device optimization, showed that upgrading to LBBP from BiV-CRT (n = 48) resulted in significant improvements in heart function as assessed by echocardiography and better clinical outcomes compared to remaining in BVP (n = 97) [92]. Similarly, a randomized trial by Wang et al. comparing LBBP and Biv-CRT in non-responders after six months of optimal treatment demonstrated a greater improvement in EF with LBBP in HF patients with NICM and LBBB [93].

Additionally, systematic reviews including HF patients with QRSd > 120 ms found that LBBP reduced QRSd compared to before implantation, a marker of ventricular synchrony [94,95]. Another systematic review and meta-analysis highlighted the potential of LBBAP in patients with HF with moderately reduced EF. Ga-inYu’s study included 211 HF patients with LVEF 35–50% demonstrated that LBBAP improved systolic function and reduced QRSd in this population [96].

The LEVEL-AT trial, which aimed to compare ventricular resynchronization achieved by CSP versus BiV-CRT demonstrated comparable outcomes for LV activation time and reverse remodeling [97]. However, the study also noted higher implantation success rates of LBBAP compared to HBP (82% vs. 57%, respectively). It highlighted obstacles associated with HBP, which requires identifying the location of the His bundle and higher activation thresholds. In comparison, LBBAP maintains advantages over HBP, like a lower and more stable capture threshold, as well as a faster left ventricular activation time. Similar results were also found in a network meta-analysis by Mariani et al. comparing different pacing techniques such as HBP, LBBAP, right ventricular pacing, and BiV-CRT published in 2023. LBBAP was found to have significantly improved LVEF, reduced HF hospitalizations, and narrowed QRSd compared to other modalities [98]. Furthermore, a review by Diaz et al. published in 2023 found that LBBAP improves the quality of life and functional status of patients who have persistent congestive heart failure despite maximal medical therapy [99].

In summary, LBBAP is emerging as a promising strategy for patients who do not respond to conventional BiV-CRT, offering improved cardiac synchronization and better clinical outcomes in various subsets of HF patients.

## 6. A Route Revisited: Endovascular Lead Placement

Although conventional CRT lead placement is usually performed through a transvenous approach via the coronary sinus, alternative surgical and endovascular techniques are utilized when the standard method proves unsuccessful [67]. Among these, endovascular lead pacing has demonstrated significant efficacy when successful. It bypasses constraints of coronary venous anatomy, mimics intrinsic endocardial-to-epicardial depolarization, and is associated with enhanced hemodynamic response [100,101,102]. It is typically implanted through subclavian or femoral venous access using a transseptal interatrial approach; however, transseptal interventricular and intra-apical approaches have also been utilized [103,104,105]. However, its utility has been limited given procedural complexities and the necessity for life-long anticoagulation with warfarin [104,106,107]. For example, while a recent systematic review of endocardial pacing noted significant improvement in registered NYHA class and QRSd in HF patients, the review also highlighted significant complication rates and a mortality rate of 20.54% at follow-up [108]. These complications ranged from infections and thromboembolic events to ischemic strokes and transient ischemic attacks.

An emerging advancement in endovascular pacing-based CRT is leadless endovascular pacing. The Wireless Stimulation Endocardially for Cardiac Resynchronization (WiSE-CRT) system is currently the only leadless endovascular pacing technology. The system includes a subcutaneous battery-connected ultrasound transmitter and an electrode implanted in the LV endocardium. It works alongside a co-implant capable of RV pacing. The transmitter detects RV pacing spikes from the co-implant and transmits ultrasonic energy which is converted by the electrode to electrical energy to pace the LV [109]. In the recently published SOLVE-CRT study, an international, multi-center, non-randomized clinical trial, 183 patients who were previously non-responders to CRT, previously untreatable, or high-risk upgrades were included [110]. The study demonstrated that leadless endocardial pacing was associated with a reduction in left ventricular ESV in patients with HF (mean percentage change of −9.3%), which the authors chose as their primary outcome given it is a well-established marker of clinical benefit in CRT [111,112,113,114]. The authors also stated that subjects had a mean absolute increase in LVEF of 5.2%. These findings suggest that leadless pacing can be a viable treatment option, especially for HF populations that are poor surgical candidates and/or previous non-responders.

## 7. Other Variations on CRT Delivery Systems

Other prominent variations on the study of optimal pacing include the use of Tri-ventricular pacing (TriV; two leads in the LV and one in the right ventricle), Adaptive CRT (CRT parameters adjust automatically with patient’s activity), and CRT fusion pacing (CRT pacing with preservation of intrinsic AV node conduction and activation via the right bundle branch) during exercise. While many comparative studies evaluating the BiV-CRT to others previously discussed have been conducted, the STRIVE HF study compared TriV pacing and found no significant difference in benefit to HF patients [115]. Another study focused on whether adaptive CRT or continuous automatic optimization stimulating only the left ventricle to fuse with intrinsic right bundle conduction was superior to conventional CRT. However, the results suggested that adaptive CRT did not significantly reduce the incidence of all-cause death or intervention for HF decompensation in patients with heart failure, left bundle branch block, and intact AV conduction [116]. The variety of new and developing pacemaker delivery systems, along with electrical conduction abnormalities among HF patients, remains a topic for further research and treatment optimization.

## 8. CRT for Special Populations

In recent years, while the growth of CRT research has largely focused on optimal pacemaker selection, leadless pacing, image-guided pacing, and CRT-D, other studies consider contextual factors including patient demographics, supplemental treatment, lead number, and fusion pacing. For example, one study compared the effectiveness of CRT between men and women to determine why women were more prone to successful fusion with intrinsic conduction. Results suggested an association with the shorter PR intervals and QRSd, more commonly associated with women than men [117]. Other studies comparing CRT effectiveness between sexes found that QRSd and LVEDV, which were more likely to be shorter and smaller, respectively, were greater determinants of successful CRT than sex, as women with greater QRSd were at risk of relative dyssynchrony [118].

Given the complex interplay between the renal and cardiovascular system, many patients with chronic kidney disease (CKD) develop cardiovascular disease, including HF. In a meta-analysis involving more than a million patients with HF, Damman et al. found that 49% of HF patients had concomitant CKD [119]. Many studies have evaluated the role of CRT for HF patients with CKD [120]. Current evidence suggests that, while reverse myocardial remodeling is observed in all stages of CKD, it is more pronounced in those with early-stage CKD [121,122,123]. Moreover, baseline renal function has shown to be an independent predictor of echocardiographic response, as well as of mortality [121,122]. Interestingly, renal responsiveness, or improvement of estimated glomerular filtration rate (eGFR) from baseline rate, was also associated with reduced risk of mortality and progression of HF, in contrast to those without eGFR improvement [121,123,124]. Furthermore, the value of eGFR 6 months post-CRT was considered an independent predictor of mortality in advanced HF patients [124,125]. Varga et al. hypothesize that outcomes regarding the association of eGFR improvement following CRT implantation signify that the improved cardiac function leads to increased renal perfusion and filtering capacity [120].

For CKD patients who are, or are expected to become, dialysis-dependent, consideration must be taken regarding the availability of vascular site access and frequency of fistula revisions. Moreover, these patients have shown to have higher odds of postoperative hemorrhage, mechanical complications, prolonged hospitalizations and increased in-hospital mortality compared to other patients [126,127]. Studies have also shown that incidence rates of DRI are higher in ESRD patients [128,129]. In this patient population, placement of transvenous leads contralateral to the fistula site is recommended, if feasible, to reduce rates of symptomatic central vein stenosis [130]. Further research is warranted, including the efficacy of leadless CRT devices, given the increased complication rates in this population group.

Regarding CRT in elderly populations, a retrospective study by Safdar et al. found that patients aged 80 or older had a similar echocardiographic response to CRT as compared to patients less than 70 [131]. These findings were paralleled by a long-term outcome study involving 2256 patients by Behon et al. [132]. Nonetheless, both studies found lower survival rates in the older groups, partially attributable to higher comorbid status [131,132]. Interestingly, a meta-analysis by Zeitler et al. highlighted that when age was treated as a continuous variable, as opposed to a categorical one (<70 years of age versus >70 years old), older individuals exhibited a greater benefit from CRT regarding combined endpoint for all-cause mortality or HF hospitalization [133]. An additional consideration in elderly populations with CRT-D is long-term goals of care. As cognitive ability declines with age, patients’ ability to make informed decisions regarding device therapy becomes a challenge. In the absence of clear goals of care and advance directives, elderly patients with cognitive impairment may be subject to unnecessary shocks and other device-related complications. One study in men over the age of 80 showed that, while the majority of elderly ICD patients had advance directives in place, a significant minority did not [134]. Ideally, conversations regarding advance directives, power of attorney, and medical order for life-sustaining treatment should take place at the time of device implantation while patients have decision-making capacity. This way, clear goals regarding criteria for device deactivation can be set, and patients will avoid unnecessary shocks at the end of life. These discoveries suggest potential areas of further research as well as information affecting future treatment guidelines based on clinical and demographic factors.

Although many studies analyzing the utility of pacemakers to treat HF along with aberrant arrhythmias such as AF are relatively common, the treatment of other chronic illnesses in patients with HF was also studied in conjunction with CRT. In the IRON-CRT trial, HF patients with iron deficiency anemia who were treated with ferric carboxymaltose were more likely to experience improvements in their LVEF, LVESV, and cardiac force-frequency relationship compared to placebo [135]. The APAF-CRT studied pharmacologic rate control in HF patients with AF, finding that those who experienced cardiac ablation and subsequent CRT demonstrated reduced mortality than those treated with pharmacological rate control followed by CRT [136]. This study demonstrates the benefits and limitations of pharmacological intervention as an adjunct to CRT.

## 9. Emerging Therapy—Cardiac Contractility Modulation

In recent years, contractility modulation (CCM) devices have been gaining interest for patients with HF. Implanted similarly to CRT, CCM devices consist of a pulse generator and two guide wires implanted into the RV septum [137]. The device delivers high-voltage electrical signals during the absolute refractory period of cardiomyocyte action potential, leading to an increase in intracellular calcium with resulting stronger myocardial contraction [138]. While the mechanism behind its beneficial effects is still being explored, multiple studies have demonstrated its efficacy in improving functional status and LVEF as well as reducing hospitalizations and cardiovascular death [139,140,141]. It is currently FDA approved for patients with NYHA class III with LVEF 25–45% who are not candidates for CRT [10].

In 2007, Butter et al. performed the first study of CCM in a patient previously failing CRT, and showed that its implantation is feasible and that CCM and CRT devices can co-exist [142]. In 2008, Nagele et al. studied CCM in 16 CRT nonresponders, and found LVEF increased and NYHA functional class improved at follow up [143]. Similarly, Kuschyk et al. in 2019 included 17 patients with HFrEF and previous non-response, and found that it improved exercise tolerance, quality of life, NYHA classification and LVEF [144]. These data sets highlight the potential of CCM as an effective therapy option for those who do not respond to CRT, and further studies are warranted to evaluate the potential role of CCM for non-response patients.

## 10. Conclusions

Non-response among CRT patients remains a significant challenge in the management of HF, particularly in individuals at high risk for arrhythmias. To address this issue, recent advancements in CRT technology have introduced alternative modalities with the potential to enhance outcomes. CSP devices such as LBBP-CRT have emerged as a promising innovation for the future of pacing, demonstrating superior improvement in cardiac function. Other exciting new strategies continue to garner attention, including image-guided placement, remote monitoring of devices, endovascular lead placement, and MPP. Future CRT research will likely focus on optimizing long-term outcomes and further refining device selection criteria. Other identified gaps in the 2023 HRS guidelines on cardiac physiologic pacing include long-term effects of CSP, CRT outcomes in patients with QRS < 150 ms, replacement/upgrade considerations in CSP, and manufacturer development of CSP-specific devices and leads [24]. Despite these advancements, patient care should remain personalized, emphasizing a tailored approach in which electrophysiologists select CRT devices based on individual risk profiles, clinical characteristics, and established guidelines. By balancing innovation with patient-centered care, CRT can continue to evolve as a cornerstone therapy for optimum HF management.

## Figures and Tables

**Table 1:** Current Guidelines and Recommendations for the use of Cardiac Resynchronization Therapy Per 2022 AHA/ACC/HFSA Guideline For the Management of Heart Failure. CRT: Cardiac Resynchronization Therapy; LVEF: Left Ventricular Ejection Fraction; GDMT: Guideline-Directed Medical Therapy; AV: Atrioventricular; LBBB: Left Bundle Branch Block; NYHA: New York Heart Association; AF: Atrial Fibrillation; QRSd: QRS duration.

Class 1	LVEF ≤ 35%, sinus rhythm, LBBB with QRS >150 ms NYHA and class II, III, or ambulatory IV symptoms on GDMT
Class 2a	LVEF ≤ 35%, sinus rhythm, non LBBB with QRS >150 ms NYHA and class II, III, or ambulatory IV symptoms
Class 2a	High-degree/complete heart block and LVEF of 36–50%
Class 2a	LVEF ≤ 35%, sinus rhythm, LBBB with QRS duration of 120–149 ms NYHA class II, III, or ambulatory IV symptoms on GDMT
Class 2a	AF and LVEF ≤ 35% on GDMT, CRT can be useful if: ○ the patient requires ventricular pacing or otherwise meets CRT criteria ○ AV nodal ablation or pharmacological rate control will allow near 100% ventricular pacing with CRT.
Class 2a	LVEF ≤35% and undergoing placement of a new or replacement device implantation with anticipated requirement for significant (>40%) ventricular pacing
Class 2b	LVEF ≤ 35%, sinus rhythm, a non-LBBB pattern with QRSd of 120 to 149 ms NYHA class III or ambulatory class IV on GDMT
Class 2b	For patients who have LVEF ≤ 30%, ischemic cause of HF, sinus rhythm, LBBB with a QRSd of ≥ 150 ms, NYHA class I symptoms on GDMT
Class 3 No benefit	If QRSd < 120 ms, CRT is not recommended.
Miscellaneous	If NYHA class I or II symptoms and non-LBBB pattern with QRSd < 150 ms, CRT is not recommended.
Miscellaneous	If comorbidities or frailty limit survival with good functional capacity to <1 year, ICD and CRT with defibrillation are not indicated.

**Table 2:** Landmark Trials in Cardiac Resynchronization Therapy for Heart Failure. CRT: Cardiac Resynchronization Therapy; CRT-P: CRT-pacemaker; CRT-D: CRT-Defibrillator; LVEF: Left Ventricular Ejection Fraction; OMT: Optimal Medical Therapy; NYHA: New York Heart Association; QOL: Quality of Life; BiV-CRT: Biventricular CRT; QRSd: QRS duration; 6MWT: 6 min walk test.

Study	Study Population and Randomization	Summary of Findings
MIRACLE Abraham et al., 2002 [30]	453 patients with LVEF ≤ 35%, NYHA class III or IV, 6MWT < 450 m, and QRS interval > 130 ms Randomly assigned to CRT group or to control group; followed for 6 months.	CRT group had improved 6MWT, EF, decreased hospitalization rates versus control group at 6-month follow up.
COMPANION, Bristow et al., 2004 [29]	1520 patients with LVEF ≤ 35%, NYHA class III or IV and QRSd > 120 ms. Randomized in 1:2:2 ratio to receive OMT alone or in combination with CRT-P or CRT-D; followed for 15 months.	CRT with or without ICD was associated with 1-year relative risk reduction of about 20% for all-cause death or hospitalization.
REVERSE Linde et al., 2008 [31]	610 patients with LVEF ≤ 40%, NYHA class I or II, QRS ≥ 120 ms. Randomly assigned to active CRT group or control group, both receiving OMT; followed for 12 months.	CRT group had reduced risk for HF hospitalization, improved ventricular structure, and NYHA I and II class.
MADIT-CRT Moss et al., 2009 [32]	1820 patients with LVEF ≤ 30%, QRS ≥ 130 ms, NYHA class I or II. Randomized in 3:2 ratio to receive CRT-D or ICD alone. Mean follow-up 2.4 years	CRT-D group had decreased mortality and CHF events when compared to ICD alone group.
CARE-HF, Cleland et al., 2005 [33]	813 patients with LVEF ≤ 35%, QRS ≥ 120 ms, NYHA class 3 or 4 despite OMT. Randomized to undergo BiV-CRT or medical therapy alone. Mean follow-up 29.4 months.	The BiV-CRT group had improved symptoms, QOL, less complications, and improved mortality. Broader QRS patients had overall better results.
RAFT, Tang et al., 2010 [34]	1798 patients with LVEF ≤ 30%, QRS ≥ 120 ms, NYHA class II or III. Randomized to obtain CRT-D or ICD alone. Mean follow up of 40 months.	CRT-D decreased mortality when compared to ICD implantation alone (though with greater adverse effects).

**Table 3:** Potential Shortcomings and Complications of CRT Devices. CRT indicates Cardiac Resynchronization Therapy.

Drawbacks of CRT	
Procedural Complications	Factors Reducing Response to CRT
Lead-related issues (i.e., malfunction or dislodgement)	QRS of less than 150 ms
Infection	Not optimized medical therapy
Hematoma	No myocardial viability at paced site
Device malfunction	Significant presence of scars

**Table 4:** Comparison of His-Bundle Pacing (HBP) and BiV-CRT (Biventricular Cardiac Resynchronization Therapy).

Type of Pacing	Lead Placement	Mechanism of Action	Advantages	Disadvantages
Biventricular CRT (BiV-CRT)	Apex of the right ventricle and lateral left ventricular wall	An electrical impulse simultaneously stimulates both the right and left ventricle to contract	Can bypass physical barriers (e.g., scarring) that impede the electrical conduction pathway	Non-physiological stimulation (extrinsic activation via the epicardium instead of the epicardium) Requires locating the optimal left ventricular pacing site to increase response
His-bundle Pacing (HBP)	Within the membranous interventricular septum, approximately near the superior border of the tricuspid valve annulus	An electrical impulse stimulates the area approximately below the Bundle of His, eliciting a signal to the remainder of the electrical conduction pathway	Mimics the heart’s natural electrical conduction pathway Shown to improve left ventricular ejection fraction and reduce QRS duration better than BiV-P	Cannot overcome electrical impedance below the atrioventricular (AV) node (i.e., Infra-Hisian blocks) Requires a greater electrical impulse (i.e., high capture threshold) for stimulation Requires precise lead implantation; more technically challenging

## Data Availability

All obtained data are included in the manuscript.
